# Ginger Extract Inhibits Biofilm Formation by *Pseudomonas aeruginosa* PA14

**DOI:** 10.1371/journal.pone.0076106

**Published:** 2013-09-27

**Authors:** Han-Shin Kim, Hee-Deung Park

**Affiliations:** School of Civil, Environmental and Architectural Engineering, Korea University, Anam-Dong, Seongbuk-Gu, Seoul, South Korea; University of Malaya, Malaysia

## Abstract

Bacterial biofilm formation can cause serious problems in clinical and industrial settings, which drives the development or screening of biofilm inhibitors. Some biofilm inhibitors have been screened from natural products or modified from natural compounds. Ginger has been used as a medicinal herb to treat infectious diseases for thousands of years, which leads to the hypothesis that it may contain chemicals inhibiting biofilm formation. To test this hypothesis, we evaluated ginger’s ability to inhibit *Pseudomonas aeruginosa* PA14 biofilm formation. A static biofilm assay demonstrated that biofilm development was reduced by 39–56% when ginger extract was added to the culture. In addition, various phenotypes were altered after ginger addition of PA14. Ginger extract decreased production of extracellular polymeric substances. This finding was confirmed by chemical analysis and confocal laser scanning microscopy. Furthermore, ginger extract formed noticeably less rugose colonies on agar plates containing Congo red and facilitated swarming motility on soft agar plates. The inhibition of biofilm formation and the altered phenotypes appear to be linked to a reduced level of a second messenger, bis-(3′-5′)-cyclic dimeric guanosine monophosphate. Importantly, ginger extract inhibited biofilm formation in both Gram-positive and Gram-negative bacteria. Also, surface biofilm cells formed with ginger extract detached more easily with surfactant than did those without ginger extract. Taken together, these findings provide a foundation for the possible discovery of a broad spectrum biofilm inhibitor.

## Introduction

Most bacterial communities grow in 3-dimensional biofilm structures on surfaces in natural, clinical, and industrial settings [1]. Biofilms consist of a single or multiple species of bacteria that are imbedded in an extracellular polymeric substance (EPS) composing of polysaccharides, proteins, and nucleic acids [2]. EPS attaches biofilm cells firmly to surfaces and protects them from harsh conditions. One noticeable feature of biofilm cells is increased resistance to detergent or biocides [3]. Possible reasons of this feature that may be due to the EPS layer include the limitation of the transport of the agents to interior bacterial cells in thick layers [4,5] and the reduction of available agents by adsorption into or reaction with the EPS matrix [6]. The ineffectiveness of antibiotic treatment in the biofilm diseases may cause serious problems in the eradication of infections [7]. In addition, biofilm formation can lead to substantial economic losses in engineering systems [8] owing to corrosion, reduced heat transfer, and increased friction.

Compounds that kill or inhibit the growth of bacteria have routinely been used to interfere with biofilm formation. However, the use of these compounds may select for the strains resistant to them [9] and the application of these compounds at sub-inhibitory levels can cause biofilm stimulation [10-12]. For these reasons, inhibitors that regulate biofilm formation without interfering with bacterial growth have received attention during the last decade. Landini et al. [13] have introduced various biofilm inhibitors. Quorum sensing (QS) inhibition is the most extensively studied approach. QS is a mechanism that controls coordinated bacterial behaviors in response to the density of bacterial cells and is tightly linked to bacterial biofilm formation [14,15] as well as to the production of virulence factors [16-18]. Patulin [19], halogenated furanones [20], and analogs of 3-oxo-C12 homoserine lactone [21] are widely known to inhibit QS in Gram-negative bacteria by competing with inherent QS signal molecules (i.e., *N*-acyl homoserine lactones [AHLs]) through binding to QS signal molecule receptors, accelerating receptor turnover, and inhibiting LasR-dependent gene expression. Apart from QS inhibitors, enzymes that degrade QS signal molecules (e.g., acylase and lactonase) also interfere with bacterial QS and biofilm formation [22]. Compounds that interfere with production of bis-(3′-5′)-cyclic dimeric guanosine monophosphate (c-di-GMP) or facilitate the degradation of this molecule are another category of biofilm inhibitors. c-di-GMP is a second messenger used for signal transduction by various bacteria [23] and reportedly modulates lifestyles associated with biofilm formation [24,25]. Sulfathiazole reduces the cellular level of c-di-GMP through inhibition of c-di-GMP biosynthesis [26]. Another mechanism of biofilm inhibitors is related to the dispersal of cells from the biofilm. Some compounds such as dispersin B [27] and nitric oxide [28] reduce biofilm formation by promoting such cell dispersal. In addition to these biofilm inhibitors, bacterial capsular polysaccharides [29], triterpenes [30], DNA-degrading enzymes [31], nanoparticles [32], etc. reportedly inhibit bacterial biofilm formation.

Some biofilm inhibitors have been isolated from natural products, which is advantageous because these inhibitors are generally less toxic and more specific compared to synthetic compounds [33]. The marine seaweed *Delisea* *pulchra* produces halogenated furanones that apparently protect the seaweed from bacterial colonization by inhibiting QS [20,34]. Garlic extract has similar QS inhibitory activity as halogenated furanones [35,36] and reduces *Pseudomonas aeruginosa* biofilm formation [37]. Rasmussen et al. [37] have tested ~30 natural products and reported that some of them (e.g., bean sprout, chamomile, carrot, and garlic) display QS inhibition. Although natural products showing QS inhibition are available, those with activity associated with c-di-GMP metabolism or biofilm dispersion are rarely reported.

Ginger has been used as a culinary and medicinal herb for thousands of years [38]. A recent study has demonstrated that ginger has antibacterial activity against *Staphylococcus aureus* and *Streptococcus pyogenes* that is higher than that of commercially available antibiotics [39,40]. Park et al. [41] have isolated antibacterial alkylated gingerols from ethanol and n-hexane extracts of ginger. In addition to displaying antibacterial properties, ginger is known to have anti-tumorigenic, anti-inflammatory, anti-apoptotic, etc. characteristics [42]. However, the biofilm inhibitory effects of ginger have not been studied.

The main objective of this study was to evaluate biofilm inhibition by ginger extract. Using *P. aeruginosa* as a model biofilm-forming microorganism, we investigated the effects of ginger extract on biofilm formation using a static biofilm assay. Furthermore, we characterized ginger-treated biofilms phenotypically by EPS production, colony morphology, swarming motility, and detachment using a detergent. We found that ginger extract inhibits biofilm formation through reduction of cellular c-di-GMP.

## Materials and Methods

### Growth inhibitory test

Overnight culture of *P. aeruginosa* strain PA14 (PA14) (optical density [OD] at 595 nm = ~1.5) in AB medium (300 mM NaCl, 50 mM MgSO_4_, 0.2% vitamin-free casamino acids, 10 mM potassium phosphate, 1 mM L-arginine, and 1% glucose, pH 7.5 [43]) was diluted with fresh AB medium (1:100). The bacterial strain was then cultured, in triplicate, with ginger extract (1 and 10% (v/v) each) and without using a shaking incubator (250 rpm) at 37°C for 14 h. OD at 595 nm was measured hourly to trace bacterial growth. The growth inhibitory tests for *Escherichia coli* strain K-12 (*E. coli*), *Staphylococcus aureus* strain ATCC6538 (*S. aureus*), and *Bacillus megaterium* strain KACC91787P (*B. megaterium*) were the same as the test for PA14 except that LB was used as the growth medium.

### Preparation of ginger extract

Ginger (*Zingiber officinale*) extract was prepared according to the protocol described previously [37]. Briefly, 150 g ginger root was shredded with 300 mL toluene (99.9%) using a standard kitchen blender. After shredding, debris was allowed to settle for 24 h at room temperature. The supernatant was filtered through a Whatman no. 1 filter paper (pore size = 11 µm). Then 150 mL deionized water was added to 150 mL filtrate, and the mixture was stirred using a magnetic stirrer for 24 h at room temperature. The mixture was then left to form water and toluene phases. The water phase was collected using a pipette and filtered through a 0.22-µm micro filter (Millex^®^ filter, Carl Roth, Karlsruhe, Germany). The filtrate (100% ginger extract) was used to test whether or not ginger extract inhibits biofilm formation.

### Static biofilm formation assay

Overnight culture of PA14 (OD at 595 nm = ~ 1.5) in AB medium was diluted with fresh AB medium (1:20) with appropriate concentrations of ginger (1~10%), and the dilution (150 µL) was aliquoted onto a TPP® 96-well polystyrene microtiter plate (Sigma Aldrich, St. Louis, Missouri, USA) and incubated at 37°C for 24 h without agitation. Biofilms that formed in the wells of the microtiter plate were assayed using the method described in a previous study [44]. For the biofilm assay, OD at 595 nm of the suspended culture was initially measured. The suspended culture was then discarded and the plate was washed with phosphate-buffered saline (137 mM NaCl, 2.7 mM KCl, 10 mM Na_2_HPO_4_, 2 mM KH_2_PO_4_, pH 7.2) to remove any remaining suspended cells in the microtiter wells. The biofilm was then stained with 1.0% crystal violet for 30 min, after which the stained biofilm was washed with deionized water to remove unbound dye. The crystal violet bound to the biofilm was eluted using 100% ethanol and quantified by measuring OD at 545 nm using the iMark microplate absorbance reader (BioRad, Richmond, CA, USA) and dividing by OD at 595 nm. The static biofilm formation assay for *E. coli*, *S.* aureus, and *B. megaterium* was the same as the test for PA14 except that LB was used as the growth medium.

### EPS analysis

The EPS was extracted using a modification of the sonication method described previously [45,46]. This modification was based on the work of Vandevivere and Kirchman [47]. Briefly, planktonic samples were prepared using 5 mL overnight culture of PA14 cells (OD at 595 nm, ~1.5) with appropriate concentrations of ginger (1~10%). The cultured cells were then harvested via centrifugation at 8,000 × *g* and resuspended in 10 mL 0.01 M KCl. Biofilm samples, on the other hand, were prepared by also using the overnight culture. The aliquot was diluted with fresh AB medium (1:20) with appropriate amounts of ginger extract. Then, 3 ml of the dilution were aliquoted into borosilicate bottles and incubated at 37°C for 24 h without agitation. The suspended cultures were measured by spectrophotometer at OD 595 nm then discarded. The bottles were washed with phosphate-buffered saline (pH = 7.2) to remove any remaining suspended cells. Biofilm cells on the wall were removed by vortexing and scraping after addition of 3 ml 0.01 M KCl. The next steps in processing were the same for both planktonic and biofilm cells and are as follows. The cells were disrupted with a sonicator (VCX 750, SONICS, Newtown, CT, USA) for 4 cycles of 5 s of operation and 5 s of pause at a power level of 3.5 Hz. The sonication method did not result in significant cell lysis. This was confirmed by conducting a cell counting experiment in which the number of dead cells after sonication was less than 10% (Figure S1). The sonicated suspension was centrifuged (4,000 × *g*, 20 min, 4°C), and the supernatant was then filtered through a 0.22-µm membrane filter (Millex^®^ filter, Carl Roth). The amounts of protein and carbohydrate in the filtrate were analyzed. For the analysis of protein, 40µL filtrate was aliquoted onto a 96-well polystyrene microtiter plate, and 200 µL Lowry reagent (L3540, Sigma Aldrich, St. Louis, Missouri, USA) was added to the aliquots. After 10 min of incubation at room temperature, 20 µL Folin-Ciocalteu reagent (L3540, Sigma Aldrich) was added to the mixture. After another 30 min of incubation at room temperature, absorbance at 750 nm was measured using the iMark microplate reader. The amount of protein was quantified by dividing OD at 750 nm by OD at 595 nm.

Carbohydrate was analyzed in a manner similar to that used to quantify protein. Fifty microliters of the filtrate were aliquoted in a 96-well polystyrene microtiter plate, and 150 µL 99.9% sulfuric acid was added to the aliquots. After 30 min of incubation at room temperature, 5% phenol was added to the mixture. After another 5 min of incubation at 90°C in the water bath, absorbance at 490 nm was measured using the iMark microplate reader. The amount of carbohydrate was quantified by dividing OD at 490 nm by OD at 595 nm.

### Swarming motility assay

Swarming motility was assayed based on a previous method [48]. 10 µL of overnight culture of PA14 (OD at 595 nm, ~1.5) with ginger extract (1%) and without was spotted onto a BM-2 plate (62 mM potassium phosphate [pH 7.0], 2 mM MgSO_4_, 10 µM FeSO_4_, 0.1% casamino acid, 0.4% glucose, and 0.5% Bacto agar) and grown at 37°C for 24 h. The degree of swarming motility was evaluated by measuring the average length of produced dendrites.

### QS inhibition assay

Two genetically modified bacterial strains ( *Chromobacterium* *violaceum* CV026 [CV026] [49] and *Agrobacterium tumefaciens* NT1 [NT1] [50]) were used in the AHL-based QS inhibition assay. *Chromobacterium* *violaceum* produces a purple pigment called violacein in response to an inherent QS signal molecule (*N*-hexanoyl-L-homoserine lactone [HHL]). CV026 is the mini-Tn5 mutant of *C* *. violaceum* that cannot produce violacein without exogenous addition of HHL. Interestingly, CV026 produces violacein with exogenous addition of various AHLs (e.g., *N*-butanoyl-L-homoserine lactone [BHL]) as well as HHL. This feature of CV026 is frequently exploited to assay QS inhibition [51,52]. Conversely, NT1 has plasmids containing the *lacZ* reporter gene merged with the *traR* gene. NT1 is known to turn blue in the presence of various AHLs including *N*-3-oxododecanonyl-L-homoserine lactone (OdDHL) and *N*-3-oxohexanoyl-L-homoserine lactone (OHHL) [53]. Overnight culture of CV026 or NT1 was diluted with fresh Luria-Bertani medium (1:20), and the dilution (115 µL) was aliquoted onto a 96-well polystyrene microtiter plate with 15 µL X-gal (50 mg/mL) (X-gal was added to NT1 but not to CV026), 5 µL AHL, and 15 µL ginger extract (0–10%) or 1 mM 2(5H)-furanone (Sigma Aldrich, St. Louis, Missouri, USA). Exogenous AHLs that lead to color changes in the QS assay for CV026 were 500 µM of BHL (BNPHARM, Daejeon, South Korea) or 20 µM of HHL (BNPHARM), whereas those for NT1 were 1 µM of OdDHL (Sigma Aldrich) or 1 µM of OHHL (BNPHARM). The mixture was then incubated at 30°C for 2 days. QS inhibition was assayed by measuring OD at 545 nm and 590 nm for NT1 and CV026, respectively, using the iMark microplate absorbance reader.

### Colony morphology assay

Overnight culture of PA14 (OD at 595 nm = ~1.0) in a T-broth medium (10 g/L tryptone) with ginger extract (1%) and without (2 µL each) was spotted onto Congo red plates (10 g/L tryptone, 40 µg/mL Congo red, 20 µg/mL Coomassie brilliant blue, and 1.5% Bacto agar) and incubated at room temperature for 3 days without agitation.

### Microscopy

Overnight culture of PA14 (OD at 595 nm = ~1.5) was diluted with fresh AB medium (1:20) with ginger extract (1%), and the dilution (1 mL) was seeded into a Lab-Tek II Chambered #1.5 German coverglass system (Nunc, Penfield, NY, USA) and incubated at 37°C for 24 h without agitation. The suspended culture was then discarded and washed with phosphate-buffered saline (pH 7.2) to remove suspended cells remaining in the coverglass system. Biofilm was stained using 3 staining solutions (400 µL each) for 20 minutes sequentially: DAPI (Carl Roth), fluorescein isothiocyanate-labeled type IV concanavalin A (ConA, Sigma Aldrich, St. Louis, Missouri, USA), and SYPRO Ruby (Ruby, Invitrogen, Carlsbad, CA, USA) to stain DNA, carbohydrate, and protein, respectively. The fluorophores had the following excitation and emission wavelengths (DAPI: 350 and 470 nm; ConA: 490 and 525 nm; Ruby: 470 and 618 nm). After staining with each solution, the biofilm was washed with phosphate-buffered saline. The biofilm and its matrix structure were then examined using a confocal laser scanning microscope (Carl Zeiss LSM700, Jena, Germany). Confocal images of blue (DAPI), green (ConA), and red (Ruby) fluorescence were observed simultaneously with the Z-stack mode [54-56]. Confocal images were taken with a 40× objective lens (C-Apochromat 40×/1.20 W Korr M27, Carl Zeiss) under the following conditions: X: 79.91, Y: 79.91, and Z: 1.37 µm of dimensions; 4.65 µs of pixel dwell. The images were analyzed with the Zen 2011 program (Carl Zeiss).

### Biofilm detachment assay

The biofilm detachment assay was performed using a previous method [57] with adaptations. Overnight culture of PA14 (OD at 595 nm = ~ 1.5) in AB medium was diluted with fresh AB medium (1:20) with ginger extract (1%) and without, and the dilution (150 µL) was aliquoted onto a 96-well polystyrene microtiter plate and incubated at 37°C for 24 h without agitation. 3 µL of sodium dodecyl sulfate (SDS; 10%) was then added to each well, and the mixture was incubated for 30 min. After incubation, OD at 595 nm was measured for the suspended cells. The suspended culture was then discarded and the plate was washed with phosphate-buffered saline. The steps that followed were the same as those described above for the static biofilm assay.

### Analysis of cellular c-di-GMP

c-di-GMP was analyzed using a method described previously [48]. Briefly, 37% formaldehyde (4.9 mL) was added to an overnight culture of PA14 (OD at 595 nm = ~ 1.5) with ginger extract (1%) and without. After centrifugation at 8,000 × *g* for 10 min, cells were harvested and resuspended with 40 mL phosphate-buffered saline (pH 7.2, supplemented with formaldehyde [0.18%]). After another centrifugation at 8,000 × *g* for 10 min, cells were harvested and resuspended with 5 mL deionized water and boiled for 10 min. After cooling the boiled resuspension was put on ice and the nucleotides were extracted by mixing 100% ethanol (9.3 mL). Supernatant containing the nucleotides was collected via centrifugation (10 min, 8,000 × *g*, 4°C) and lyophilized using Savant SpeedVac^®^ (SC210A, Thermo Scientific, San Jose, CA, USA). Protein in the pellet was also measured with the Lowry method described above, which was used to normalize the amount of c-di-GMP. The lyophilized supernatant was dissolved using 1 mL triethylammonium acetate and filtered through 0.22-µm syringe filter (Millex^®^ filter, Carl Roth). c-di-GMP was analyzed using triple quadruple mass spectrometry (Finnigan TSQ Quantum Ultra EMR, Thermo Scientific) coupled with high-performance liquid chromatography (Finnigan Surveyor MSQ Plus, Thermo Scientific). An Extend-C18 column (2.1 mm × 150 mm, 5-µm particle size, Agilent, Santa Clara, CA, USA) and a guard column (C18, 2.1 mm × 4 mm, Phenomenex, Torrance, CA, USA) were used for the analysis. The mobile phase consisted of 10 mM triethylammonium acetate and 10 mM acetonitrile, and the flow rate was 200 µL/min. The gradient condition was set to the following: isocratic 0% acetonitrile for 2 min, 0-20% acetonitrile for 7 min, 20-90% acetonitrile for 1 min, and isocratic 90% acetonitrile for 2 min. The injection volume of sample was 10 µL. c-di-GMP was monitored using electrospray ionization in the positive ion mode. The optimum mass spectroscopy condition was as follows: 3700 volts of spray voltage, 300°C of capillary temperature, 40 psi of sheath gas pressure, and 20 Arb of aux gas pressure, respectively. For quantitative analysis of c-di-GMP, selective reaction monitoring (m/z 691 > 152) was used. Data processing was performed using Xcalibur (Thermo Scientific).

### Statistical analysis

Statistically analyzed *P*-values were estimated by a student’s *t*-test using the Microsoft, Exel software.

## Results

### Effect of ginger extract on growth

**Figure 1:**
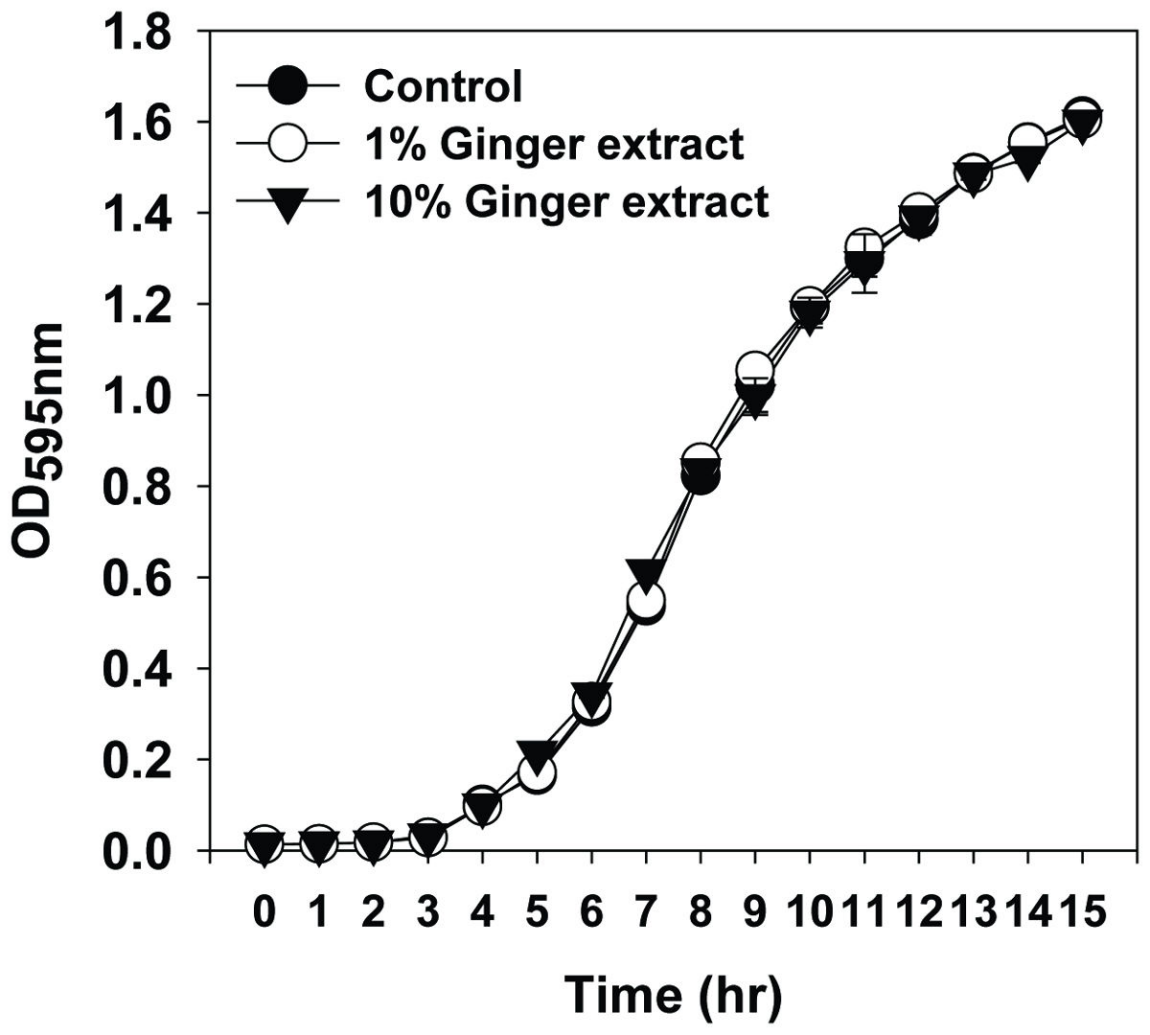
Growth curves of PA14 with 1% and 10% ginger extract. Growth curve was plotted with the control (i.e., no ginger extract addition). The growth was traced by measuring OD at 595 nm hourly for 14 h. Error bars indicate the standard deviations of 3 measurements.

To evaluate the effect of ginger extract on the growth of PA14, we grew the bacteria in batch cultures with ginger extract (1 and 10% each; Figure 1) and without. The cultures demonstrated typical bacterial growth curves including lag, exponential, and stationary phases during 14 h of incubation. In addition, no significant differences in the growth curve occurred between the cultures with ginger extract (1 and 10%) and without. The results suggested that the growth of PA14 was unaffected by the addition of ginger extract up to 10%.

### Effect of ginger extract on biofilm formation

**Figure 2:**
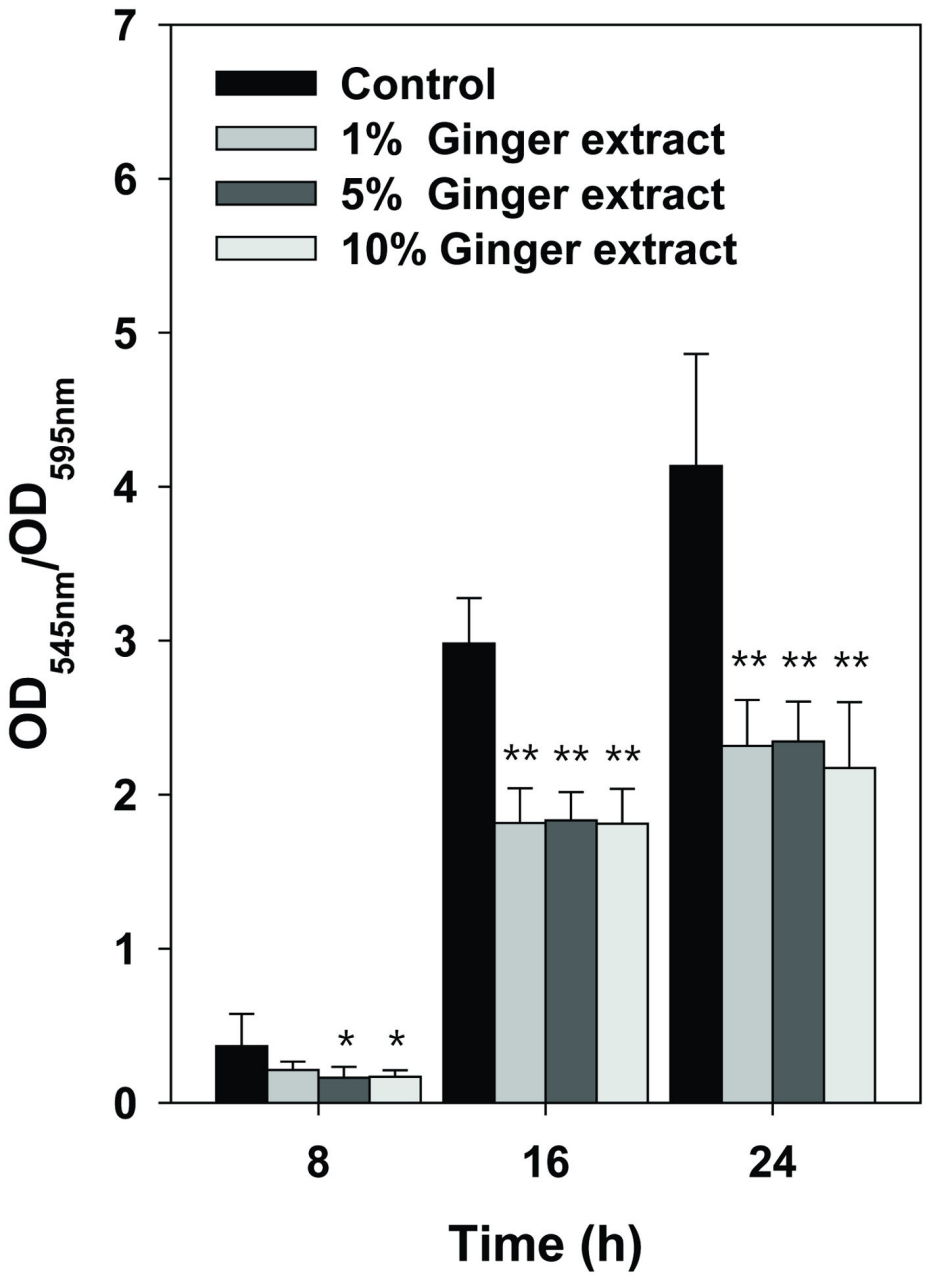
Quantification of PA14 biofilm formed in the wells of microtiter plates for cultures with various quantities of ginger extract (0, 1, 5, and 10%). The biofilm was quantified at 8, 16, and 24 h of incubation by dividing OD at 545 nm by OD at 595 nm for cells stained with crystal violet. Error bars indicate the standard deviations of 6 measurements. *, P < 0.05 versus the control. **, P < 0.00001 versus the control.

Static biofilm quantification assay was performed to evaluate the effect of ginger extract on PA14 biofilm formation. Figure 2 shows the difference in biofilm formation between the control (i.e., no ginger extract addition) and the culture containing ginger extract (1, 5, and 10% each). The microtiter plates showed that biofilm formation for the experimental group (i.e. cultures with ginger extract) was 39–56% less than the amount of formation in the control (i.e. without ginger extract). The differences in biofilm reduction for ginger extract concentration (P > 0.5) and for incubation time were not significant (P > 0.1). The results suggested that the biofilm formation of PA14 is inhibited by the addition of ginger extract in microtiter plates. The inhibition of biofilm formation by ginger extract was not due to minor contamination with toluene or something extracted from the plastic/glassware during the preparation of ginger extract, which was confirmed by an experiment using mock extraction (Figure S2).

### Effect of ginger extract on EPS production

**Figure 3:**
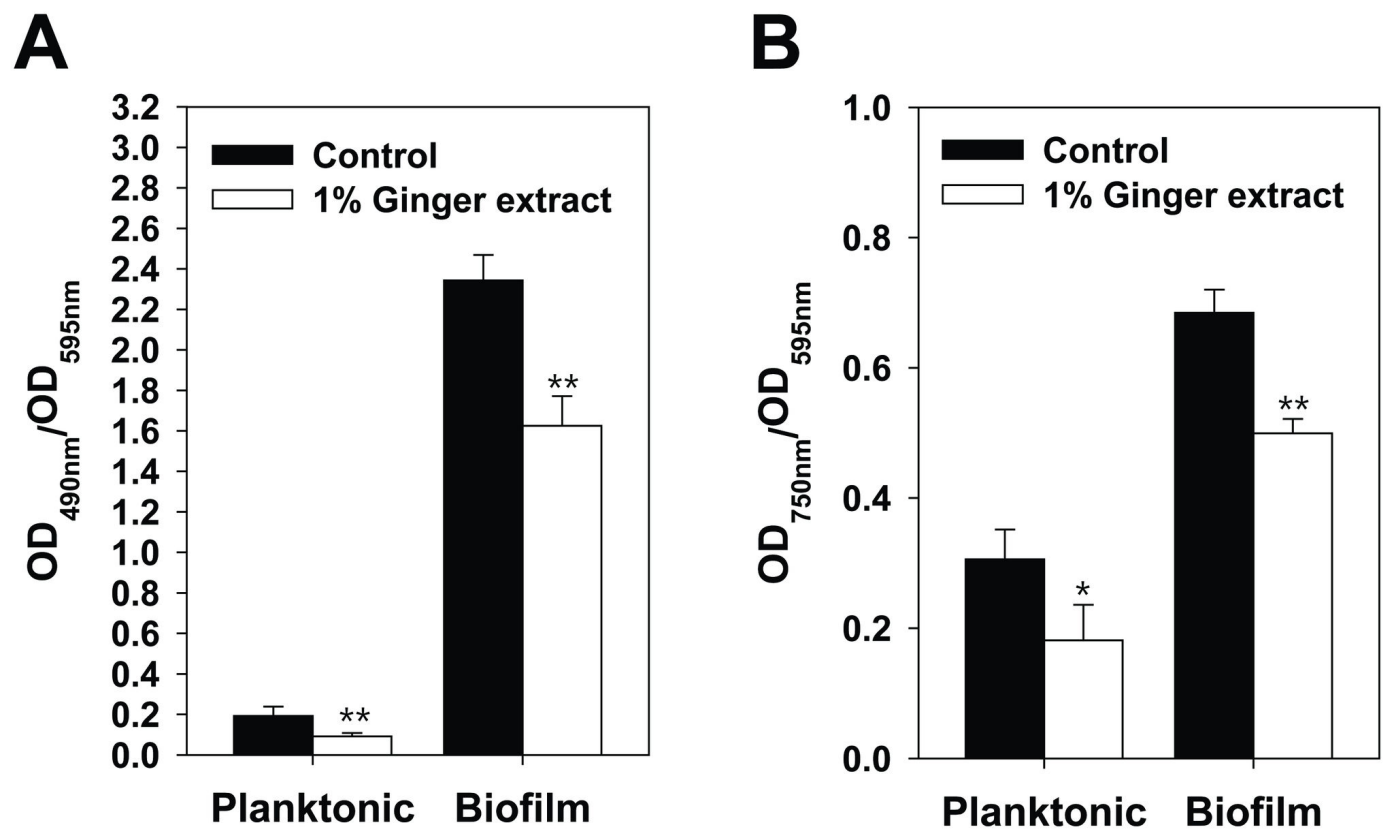
Comparison of EPS constituents of planktonic and biofilm cells with 1% ginger extract and without. (A) Total protein in EPS of PA14 cultures. (B) Total carbohydrates in EPS of PA14 cultures. Error bars indicate the standard deviations of 7 measurements. **, P < 0.001 versus the control. *, P < 0.01 versus the control.

**Figure 4:**
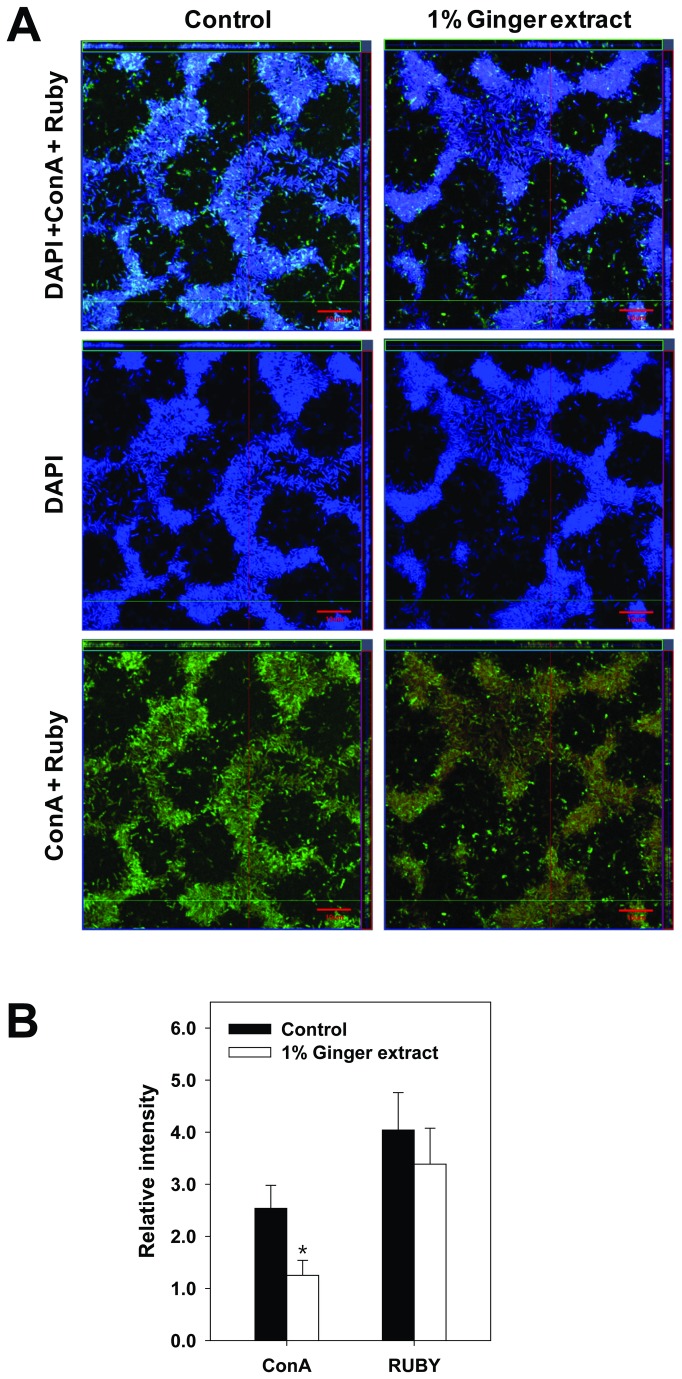
Confocal laser scanning microscope analyses. (A) Images of PA14 biofilms formed with (right) 1% ginger extract and without (left). The top images were obtained by combining DAPI, ConA, and Ruby images. The middle images are DAPI, whereas the bottom images are the combination of ConA and Ruby. The fluorescent colors generated from DAPI, ConA, and Ruby were blue, green, and red, respectively. The fluorescent color generated from the combinations of DAPI, ConA, and Ruby was cyan, while that of ConA and Ruby was yellow. (B) Relative ConA and Ruby intensities normalized by DAPI intensity for PA14 biofilms with 1% ginger extract and without. *, P < 0.01 versus the control.

To evaluate the effect of ginger extract on EPS production, we analyzed the total carbohydrate and protein content of EPS from 24-h PA14 cultures. As shown in Figure 3, both total carbohydrate and total protein were reduced by the addition of 1% ginger extract to planktonic and biofilm cells; however, total carbohydrate was reduced more than total protein. The total protein with 1% ginger addition was 59% (planktionic), and 73% (biofilm) of the control’s, whereas total carbohydrate with 1% ginger addition was 48% (planktonic), and 69% (biofilm) of the control’s. Carbohydrate and protein reductions were also detected in biofilm cells by staining biofilm cells with 3 fluorophores specific for nucleic acids (DAPI), carbohydrates (ConA), and proteins (Ruby) and imaging with a confocal laser scanning microscope. Although there were no significant difference in the number of cells between the two biofilms (Figure 4A DAPI images), the biofilm formed with ginger extract had a lower amount of EPS than that formed without ginger extract (Figure 4A ConA + Ruby images). This difference was confirmed through measurement of ConA and Ruby signal intensities normalized by DAPI signal intensity for all confocal images (Figure 4B). ConA and Ruby signal intensities for the biofilm formed with ginger extract were 49% and 84% of the signal intensity of the control (i.e., no ginger extract), respectively. There was no significant autofluorescence by PA14 cells observed in our analyses (data not shown).

### Effect of ginger extract on colony morphology on a Congo red agar plate

**Figure 5:**
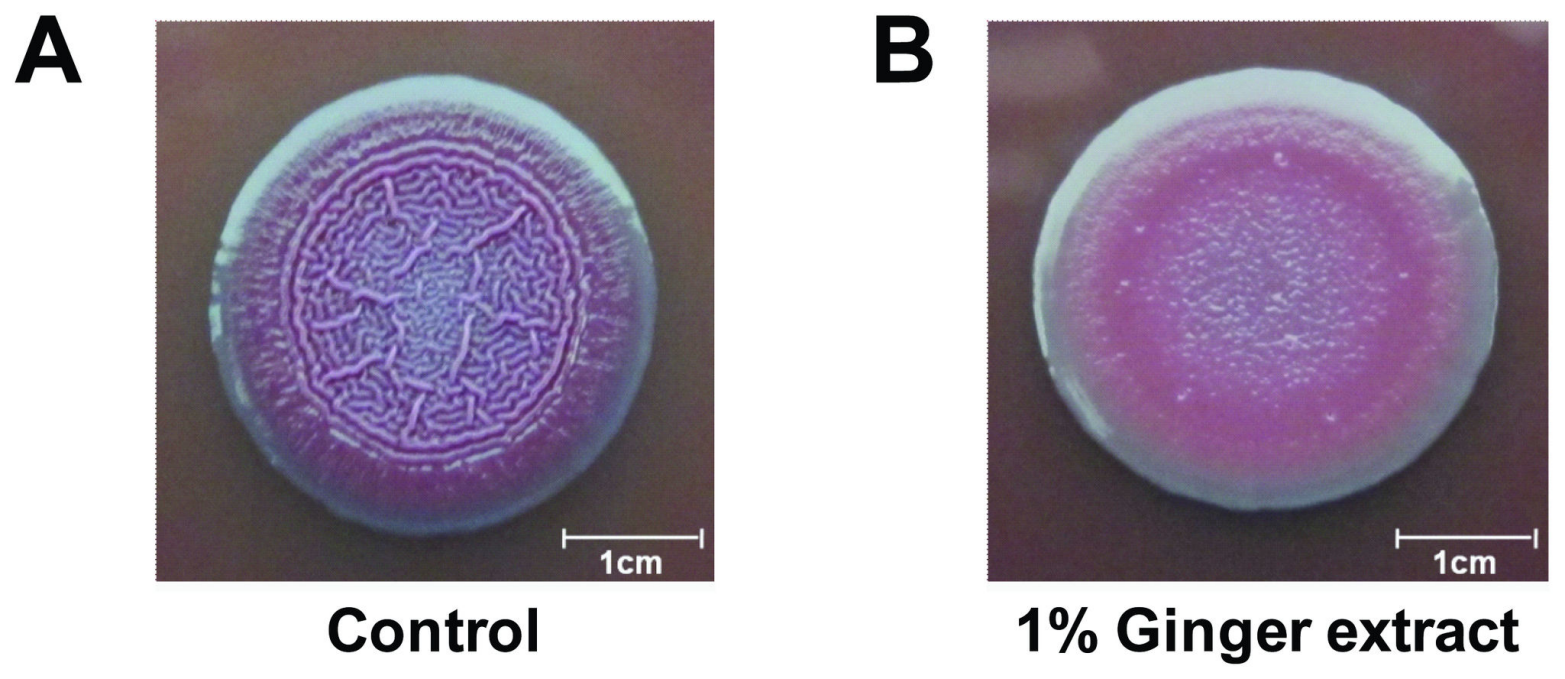
Comparison of colony morphology on Congo red agar plates. (A) Colony of PA14 grown without ginger extract. (B) Colony of PA14 grown with 1% ginger extract.

Colony morphology was observed on an agar plate containing Congo red (Figure 5). The colony grown on an agar plate without ginger extract showed a pink and rugose morphology known to be related to exopolysaccharide overproduction [48].

### Effect of ginger extract on swarming motility

**Figure 6:**
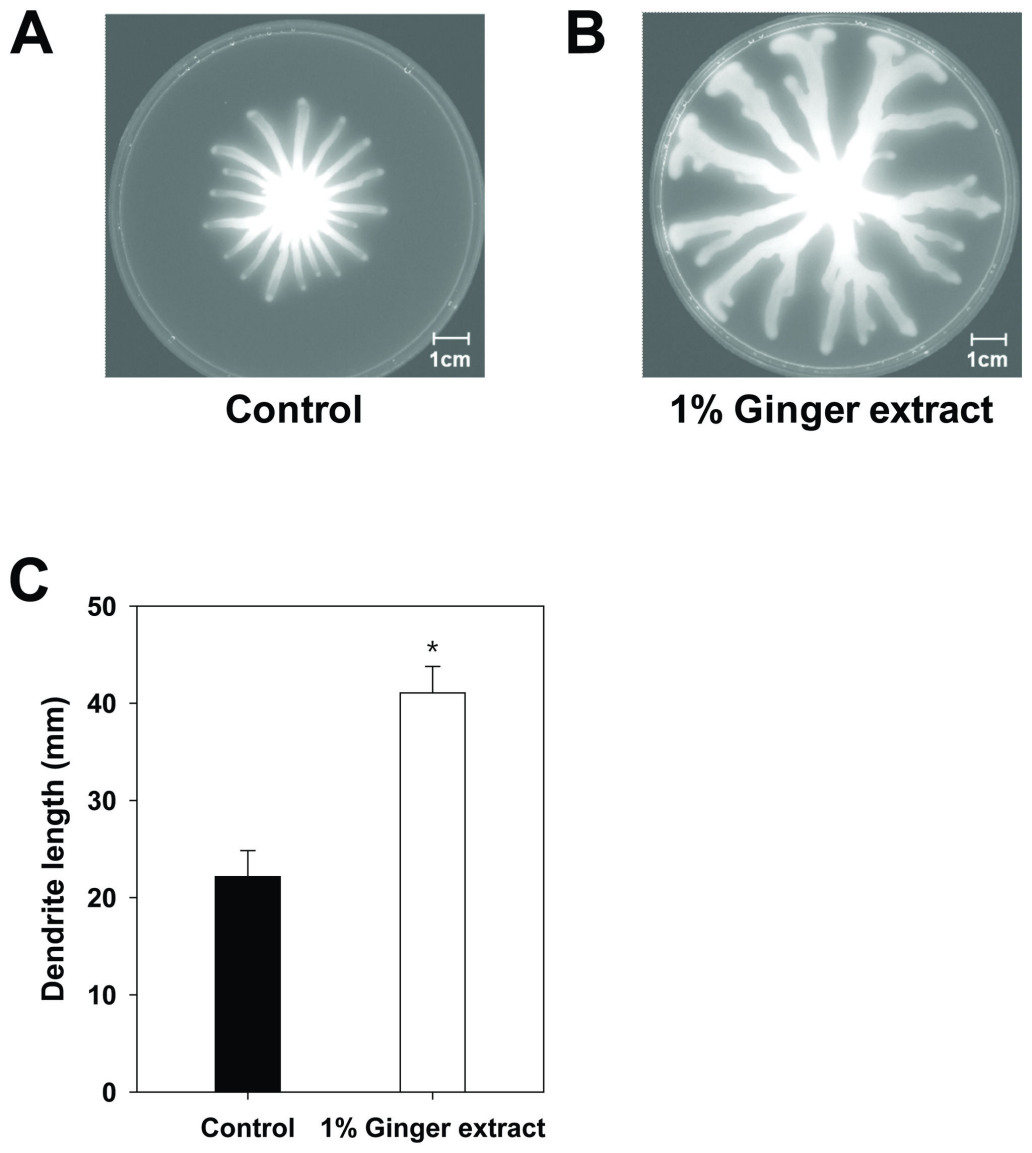
Comparison of swarming motility on swarm agar plates. (A) PA14 cells grown without ginger extract. (B) PA14 cells grown with 1% ginger extract. (C) Length of dendrites of PA14 cells grown with 1% ginger extract and without. *, P < 0.00001 versus the control.

The swarming motility of PA14 cells grown with and without ginger extract was evaluated by growing cells on a swarm agar plate and by measuring the length of dendrites from the center (Figure 6). The dendrite length of cells grown with 1% ginger extract was 43 ± 2 mm (average ± standard deviation), whereas the length for cells grown without ginger extract was 25 ± 3 mm. This result suggested that ginger extract promotes the swarming motility of PA14 on swarm agar plates.

### Effect of ginger extract on QS

**Figure 7:**
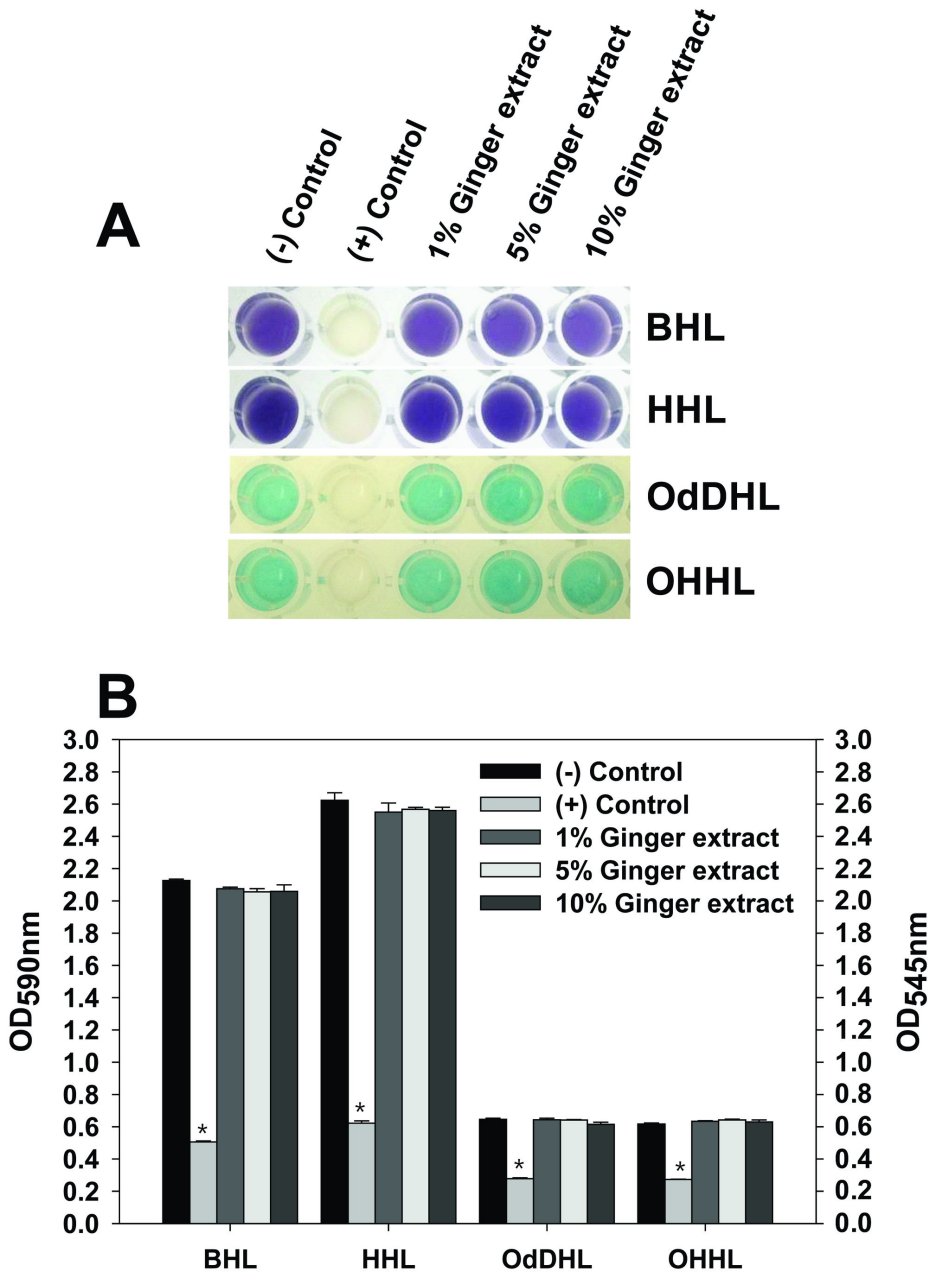
QS inhibition assay for ginger extract. QS inhibition related to BHL and HHL was assayed using CV026, whereas that related to OdDHL and OHHL was assayed using NT1. (A) Color changes of CV026 or NT1 cultures with various concentrations of ginger extract (0, 1, 5, and 10%), various AHLs (BHL, HHL, OdDHL, and OHHL) and 2(5H)-furanone (positive control) (B) Color change was measured with OD at 545 nm for NT1 cultures and with OD at 590nm for CV026 cultures, respectively. *, P < 0.0001 versus the control.

QS is often linked to bacterial biofilm formation [14]. PA14 produces 2 major (BHL and OdDHL) and 2 minor (HHL and OHHL) AHLs [58]. To examine the possibility of AHL-based QS inhibition by ginger extract in PA14 biofilm formation, we conducted a set of QS inhibition assays using CV026 and NT1 strains. CV026 was used to test QS inhibition associated with AHLs such as BHL and HHL, whereas NT1 was used to test QS inhibition associated with AHLs such as OdDHL and OHHL. The addition of ginger extract to CV026 culture did not alter the colors at any concentration (0–10%; Figure 7). Similar results were obtained for QS inhibition assays using NT1 (see Figure 7). The results demonstrated that ginger extract does not interfere with the interaction between the added AHLs and transcription factors (i.e., CviR for CV026 and TraR for NT1).

### Effect of ginger extract on the detachment of biofilm cells

**Figure 8:**
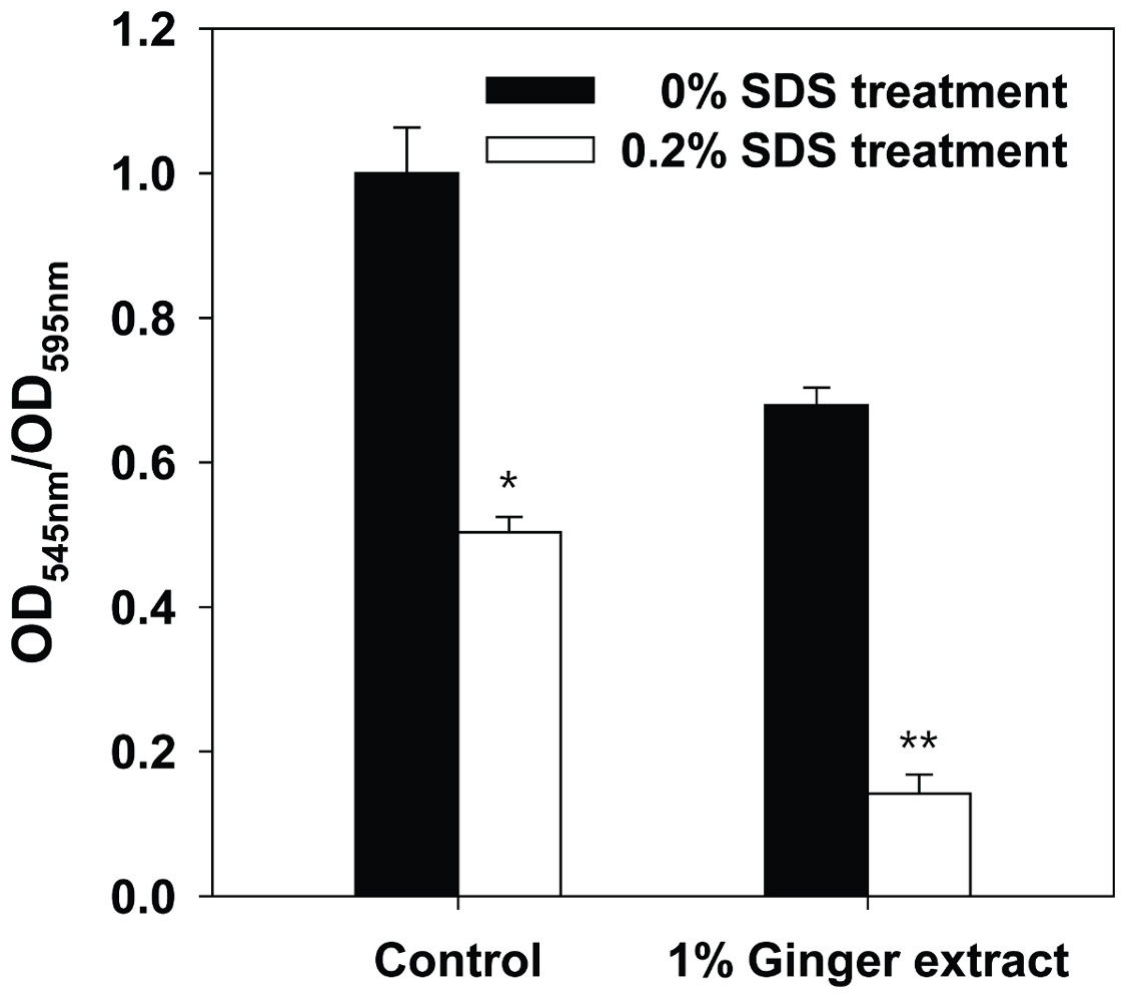
SDS detachment of PA14 biofilm formed with 1% ginger extract and without. The biofilm in the wells of microtiter plates was quantified by dividing OD at 545 nm by OD at 595 nm for cells stained with crystal violet. Error bars indicate the standard deviations of 4 measurements. *, P < 0.01 versus the control, **, P < 0.00001 versus the control.

To evaluate the effect of surfactant on the detachment of biofilm cells, we quantified the amount of biofilm after treatment with SDS in PA14 biofilms formed on the surface of microtiter plates with the addition of 1% ginger extract and without. As shown in Figure 8, SDS detached biofilm cells from both groups. However, the detachment efficiency for biofilm cells formed with ginger extract (79%) was higher than that for biofilm cells without ginger extract (50%). This result suggested that biofilm cells formed with ginger extract are more loosely attached on the surface than those formed without ginger extract.

## Discussion

This study showed that ginger extract, similar to garlic extract [37], inhibits PA14 biofilm formation without affecting the growth of bacteria (see Figure 1). This feature is important in the development of drugs used to treat biofilm-related infectious disease because it reduces the development of antibiotic-resistant bacteria [59]. Although ginger has been reported to have antibacterial activity [41], the ginger extract used in this study showed no such activity. This was possibly due to the removal of some compounds affecting PA14 growth by the extraction procedure. However, the biofilm-inhibiting mechanism of ginger extract differs from that of garlic extract. Garlic extract is reported to inhibit AHL-based QS of PA14 [37], but ginger extract showed no such mechanism, as evidenced by the QS inhibition assay (see Figure 7). In addition to its inhibition of biofilm formation, ginger extract affected various effects phenotypes of PA14. Some of these characteristics included a lowered production of EPS (see Figures 3 and 4), a noticeable reduction of rugose colonies on Congo red plates (see Figure 5), and an increase in swarming motility (see Figure 6). PA14 reportedly has genes called *pel* genes that are involved in biofilm formation both at the air-liquid interface in standing cultures (pellicle) and on surfaces [60]. These *pel* mutants cannot produce EPS or rugose colonies on agar plates containing Congo red, which binds exopolysaccharide [60]. Thus, the lack of rugose colony formation in the presence of ginger extract (see Figure 5) suggested that ginger extract reduced the production of exopolysaccharide. The measurement of total carbohydrate and protein for both suspended and biofilm cells with and without ginger extract (see Figures 3 and 4) agrees with this speculation. The increased swarming motility of PA14 associated with ginger extract can be interpreted through the findings of Caiazza et al. [61], who reported a close link between swarming motility and biofilm formation in PA14. They demonstrated an inverse regulation of biofilm formation and swarming motility of PA14 via flagella reversal and Pel polysaccharide.

**Figure 9:**
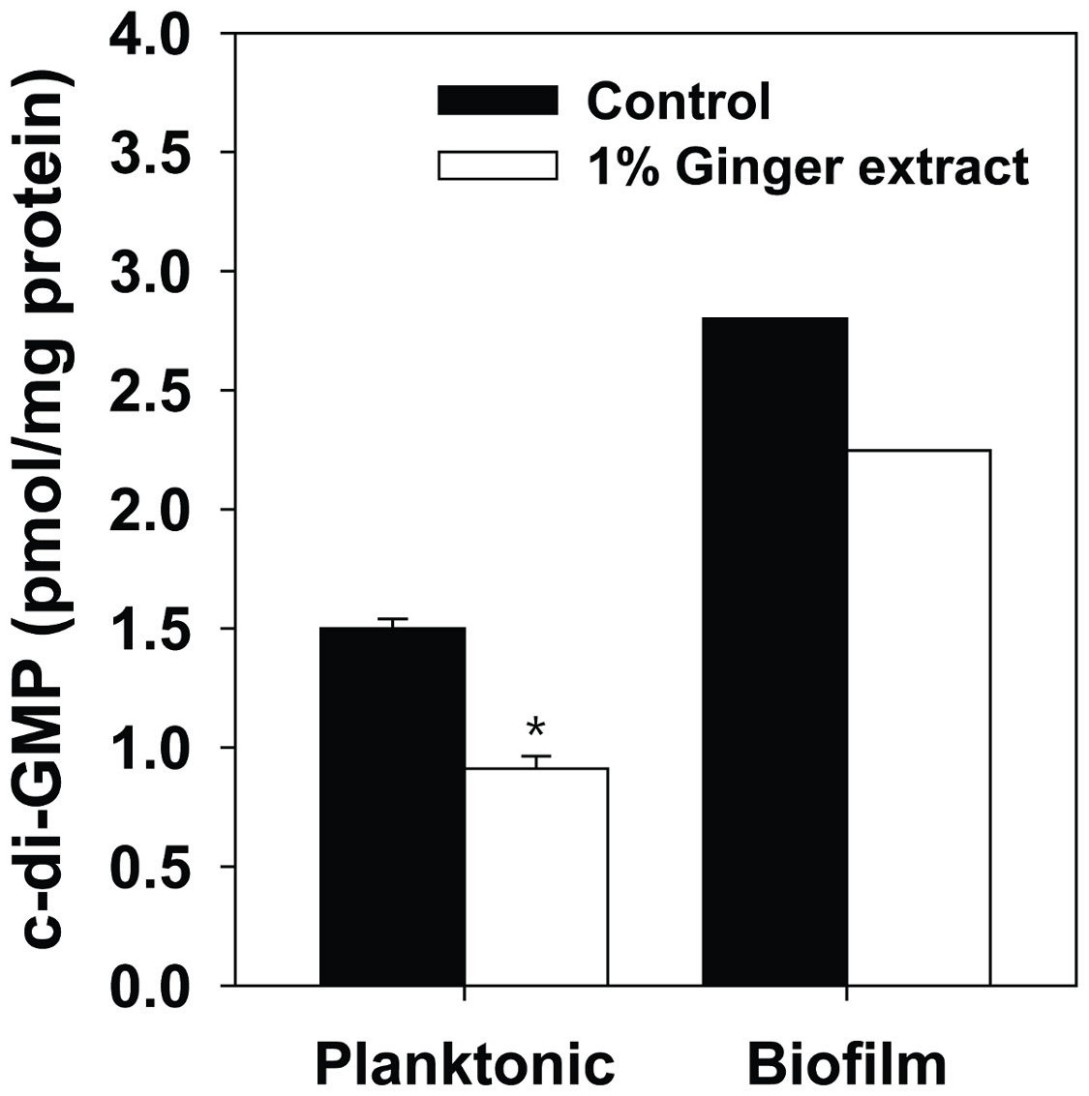
Concentration of c-di-GMP of planktonic and biofilm cells grown with 1% ginger extract and without. Error bars indicate the standard deviations of 3 measurements. *, P<0.001 versus the control. Actual mass peaks are shown in Figure S3.

According to a recent study of PA14 biofilm by Ueda and Wood [48], a battery of phenotypes of PA14, including biofilm formation, rugose colony formation, and swarming motility are regulated by the level of the signal molecule c-di-GMP. The study also showed that the level of c-di-GMP is modulated by a tyrosine phosphatase, TpbA. A reasonable hypothesis is that ginger extract inhibits PA14 biofilm formation through the regulation of cellular c-di-GMP levels. We tested this hypothesis by analyzing c-di-GMP in suspended biomass and biofilm formed with and without the addition of ginger extract. As shown in Figure 9, c-di-GMP levels of suspended biomass and biofilm formed with 1% ginger extract were 61% and 84% of the control (i.e., no ginger extract), respectively. This result suggested that ginger extract inhibited biofilm formation by lowering the level of cellular c-di-GMP in PA14. Furthermore, the addition of ginger extract reduced biofilm formation in a PA14 mutant that over-produces cellular c-di-GMP (*∆wspF*), which confirmed the relevance of ginger extract to lowering cellular level of c-di-GMP (Figure S4).

c-di-GMP was first reported by Ross et al. [62], who demonstrated that the activity of cellulose synthase in *Acetobacter* *xylinum* was regulated by this molecule. Numerous studies have demonstrated the role of c-di-GMP as a global second messenger across diverse bacteria including Gram-positive and Gram-negative bacteria, but not in archaea or eukaryotes [63]. c-di-GMP reportedly modulates bacterial physiology and behavior, including adhesion, motility, virulence, biofilm formation, and cell morphogenesis through transcription, translation, and posttranslational levels [64]. In particular, c-di-GMP is important in the molecular determination between planktonic and sessile lifestyles [65]. c-di-GMP is produced from proteins with GGDEF domains [66,67] and is degraded by proteins with EAL [67] or HD-GYP [68] domains. Interestingly, many proteins with these domains have been found both in single species as well as in diverse bacteria. For example, one genomic study [69] has shown that PA14 has 17 proteins with GGDEF domains, 5 proteins with EAL domains, and 16 proteins with both domains. Another feature of these proteins is that they have additional signal input domains that receive environmental cues. Thus, proteins can likely sense environmental cues through signal input domains and then modulate the activities of GGDEF, EAL, or HD-GYP domains, which regulates the level of c-di-GMP and in turn affects bacterial physiology and behavior. A speculated mechanism of biofilm inhibition by ginger extract is the modulation of proteins with GGDEF, EAL, or HD-GYP domains. Some compound(s) in ginger extract may increase the activity of protein(s) with EAL or HD-GYP domains, resulting in the degradation of c-di-GMP; other compounds may inhibit the activity of protein(s) with GGDEF domains, lowering the synthesis of c-di-GMP. Future study is required to elucidate the detailed mechanism through which ginger extract lowers c-di-GMP levels and to explore the active ingredients of ginger extract that affect biofilm inhibition.

**Figure 10:**
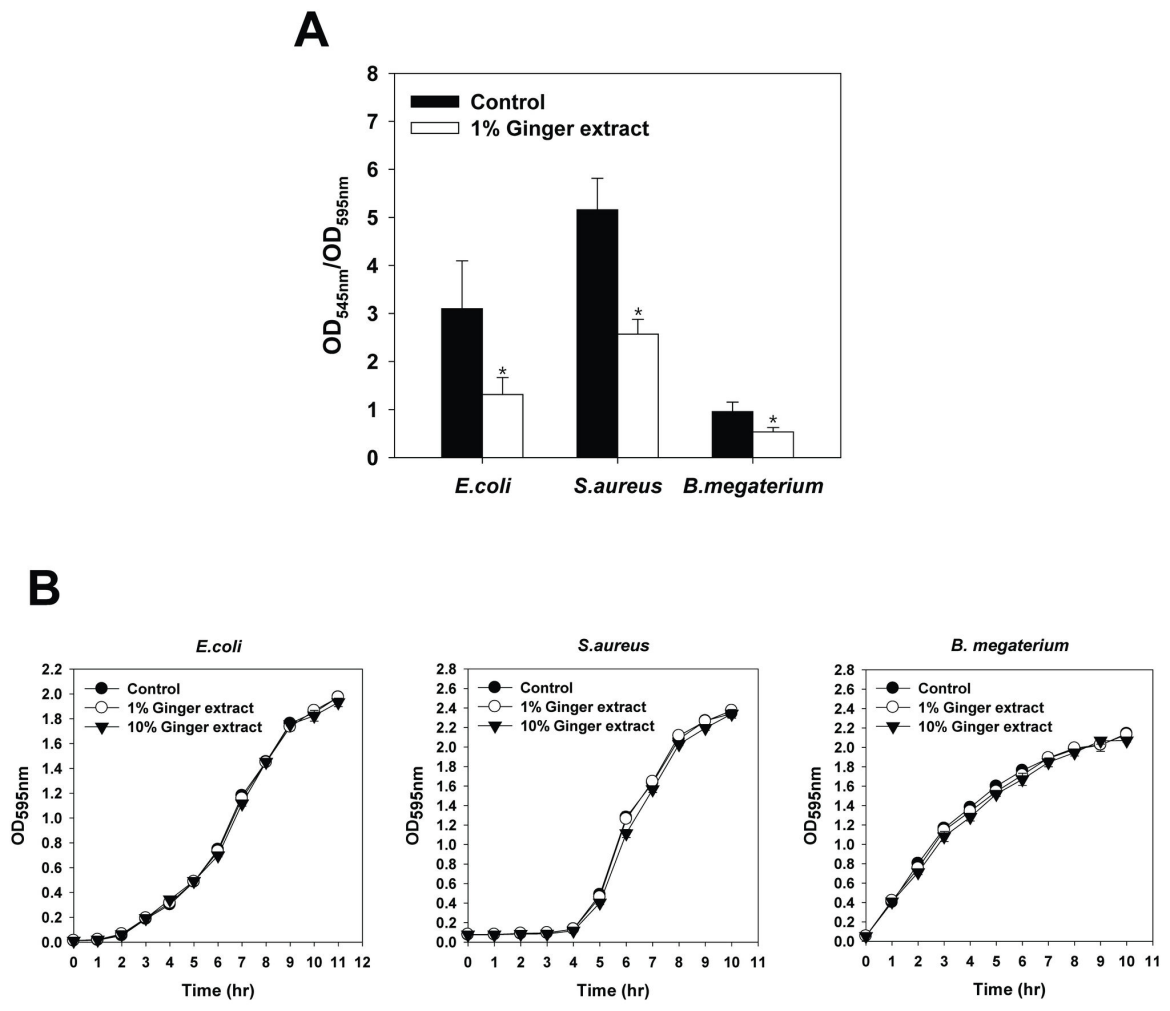
The effects of ginger extract on *E. coli*, *S.* aureus, and *B. megaterium*. (A) Biofilm formation in the wells of microtiter plates for cultures with various concentrations of ginger extract (0, 1, 5, and 10%). The biofilm in the wells of the microtiter plate were quantified at 24 h of incubation by dividing OD at 545 nm by OD at 595 nm for cells stained with crystal violet. Error bars indicate the standard deviations of 15 measurements. *, P < 0.00001 versus the control. (B) Growth curves with 1% and 10% ginger extract. Growth curve was plotted with the control (i.e., no ginger extract addition). The growth was traced by measuring OD at 595 nm hourly for 14 h. Error bars indicate the standard deviations of three measurements.

Compounds modulating the enzymes involved in biosynthesis or degradation of c-di-GMP are likely to be more influential than those targeting QS in terms of broad-spectrum biofilm inhibition [13]. Ginger extract reduces biofilm formation for various bacteria including some Gram-positive (e.g., *Staphylococcus aureus* and *Bacillus megaterium*) and Gram-negative bacteria (e.g., *Escherichia coli* and *Pseudomonas aeruginosa*) (Figure 10). Considering that most QS inhibitors are effective against either Gram-negative or Gram-positive bacteria [70], ginger extract is a promising candidate in the search for a broad-spectrum biofilm inhibitor.

Biofilm inhibitors such as ginger extract are applicable in a variety of fields. Clinically, biofilm inhibitors can be used directly to reduce virulence factors from infectious bacteria [71] or to treat infectious biofilm along with conventional antibiotics [72]. As demonstrated by the biofilm detachment with SDS (see Figure 8), biofilm inhibitors like ginger extract facilitate the separation of biofilms from surfaces. This property can be also applied industrially - for example, to the removal of biofilm formed on membrane filters for water treatment [73] or on water/oil pipes.

In conclusion, this study clearly demonstrated the effectiveness of ginger extract in inhibiting PA14 biofilm formation. Ginger extract reduced cellular c-di-GMP concentration, which appears to affect characteristic phenotypes as demonstrated by reduced biofilm formation, increased EPS production, decreased rugose colony formation on Congo red plates, and enhanced swarming motility. The results are a baseline for researching the ginger compounds involved in c-di-GMP reduction and for identifying the c-di-GMP regulation mechanisms of these compounds.

## Supporting Information

Figure S1 **Cell counts for the unsonicated suspension and sonicated suspension.** Error bars indicate the standard deviations of six measurements.
(TIF)

Figure S2 **Quantification of PA14 biofilm formed in the wells of microtiter plates for 1% mock extraction.** Control was conducted using deionized water. The biofilm was quantified at 24 h of incubation by dividing OD at 545 nm by OD at 595 nm for cells stained with crystal violet. Error bars indicate the standard deviations of 15 measurements.
(TIF)

Figure S3 **Analyses of c-di-GMP amount by LC-mass spectroscopy.** (A) Planktonic PA14 cells. (B) Biofilm PA14 cells. Data show the peaks for synthetic c-di-GMP (BIOLOGY Life Science Institute, Bermen, Germany), ethanol extract of PA14 without ginger addition (control), and ethanol extract of PA14 cultured with 1% ginger extract. c-di-GMP levels measured after about 7 minutes of retention time. The concentration of c-di-GMP was analyzed by calculating the area of peaks corresponding to the retention time.
(TIF)

Figure S4 **The effects of ginger extract on a PA14 mutant overproducing c-di-GMP (Δ*wspF*).** (A) Quantification of biofilm formed in the wells of microtiter plates for Δ*wspF* cultures with various quantities of ginger extract (0, 1, 5, and 10%). The biofilm was quantified at 24 h of incubation by dividing OD at 545 nm by OD at 595 nm for cells stained with crystal violet. Error bars indicate the standard deviations of 6 measurements. *, P < 0.05 versus Δ*wspF* (0% ginger). **, P < 0.001 versus Δ*wspF* (0% ginger). (B) Concentration of c-di-GMP for control, Δ*wspF*, and Δ*pelA*. Error bars indicate the standard deviations of 3 measurements. *, P<0.001 versus the control. (C) Actual LC-MS peaks of c-di-GMP for control, Δ*wspF*, and Δ*pelA*. Δ*pelA* is a PA14 mutant forming no pellicle.
(TIF)
