# Supplementary figures and images for: Ginger Extract Inhibits Biofilm Formation by Pseudomonas aeruginosa PA14
Source: PLoS One. 2013 Sep 27;8(9):e76106. doi: 10.1371/journal.pone.0076106 (PMC3785436; doi:10.1371/journal.pone.0076106)

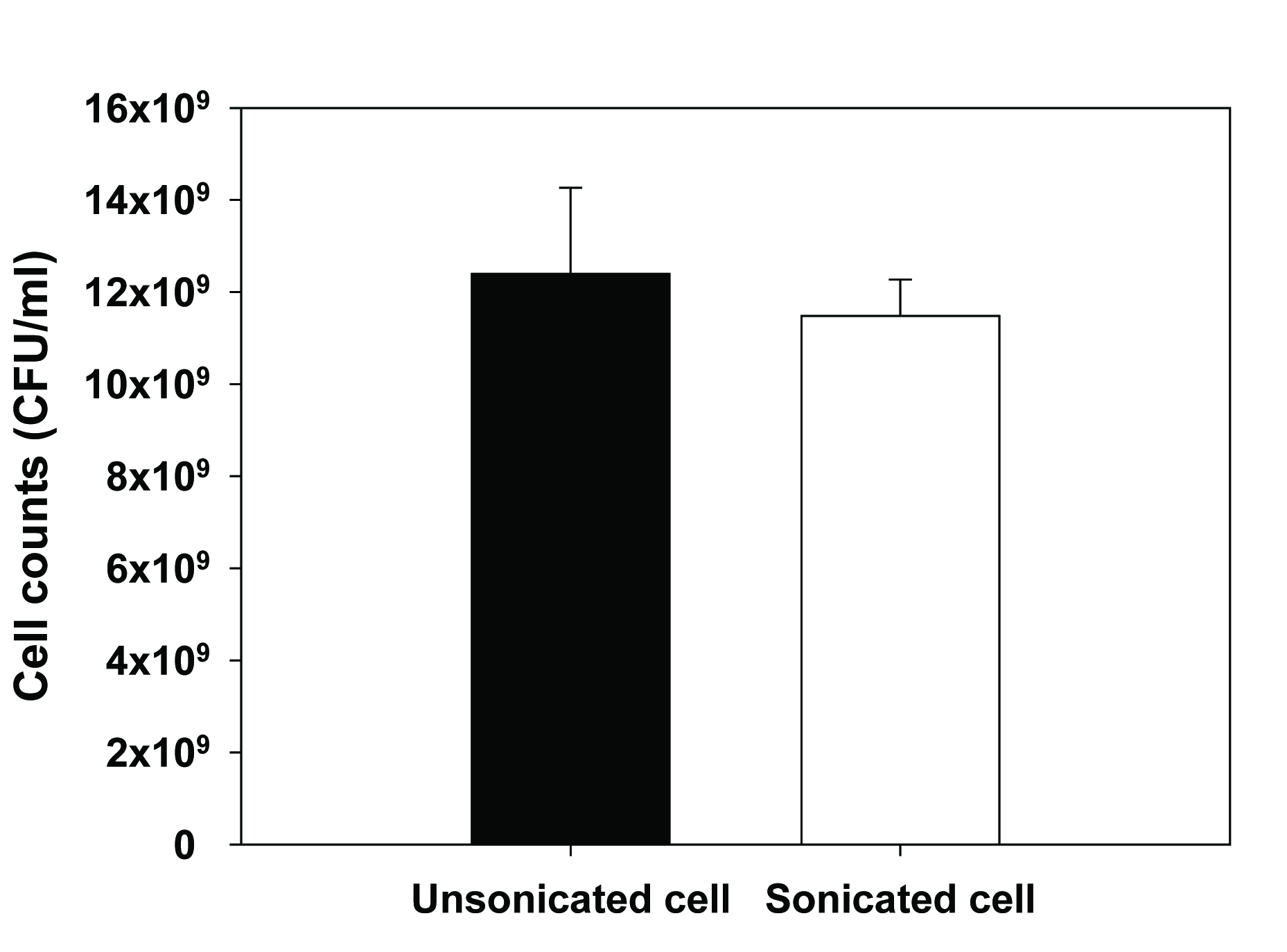

Supplement: Figure S1 — Cell counts for the unsonicated suspension and sonicated suspension. Error bars indicate the standard deviations of six measurements. (TIF) [file pone.0076106.s001.tif]

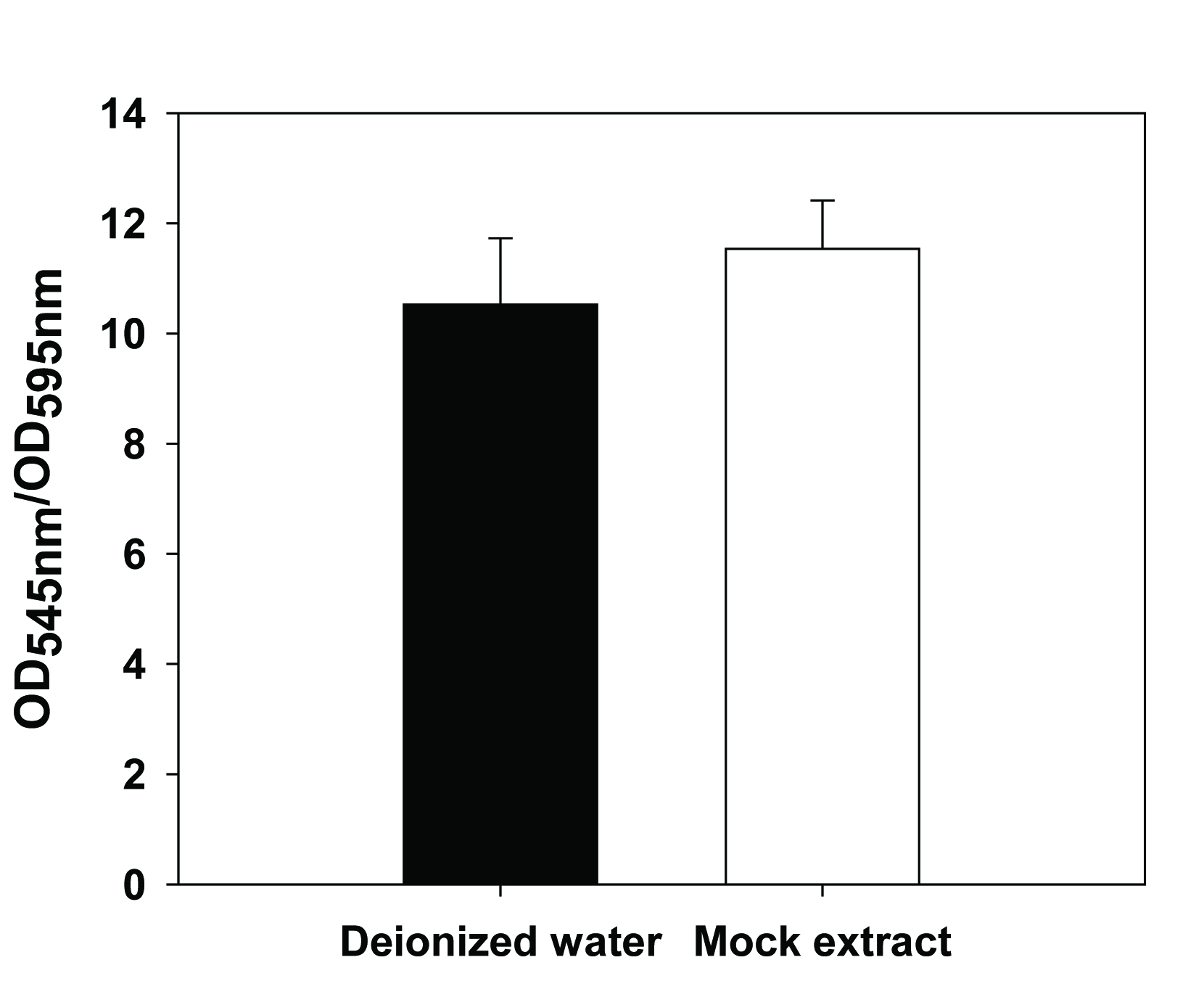

Supplement: Figure S2 — Quantification of PA14 biofilm formed in the wells of microtiter plates for 1% mock extraction. Control was conducted using deionized water. The biofilm was quantified at 24 h of incubation by dividing OD at 545 nm by OD at 595 nm for cells stained with crystal violet. Error bars indicate the standard deviations of 15 measurements. (TIF) [file pone.0076106.s002.tif]

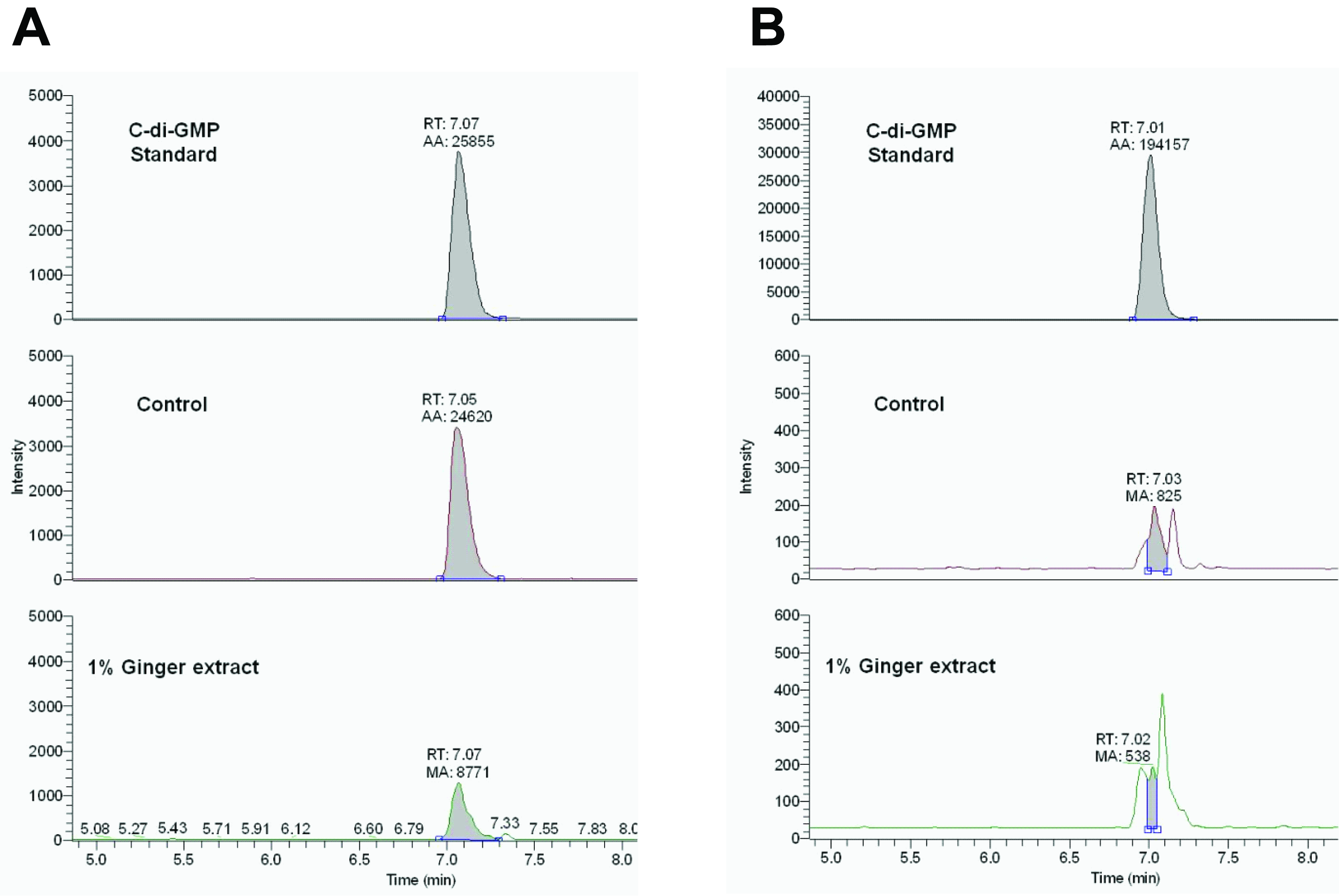

Supplement: Figure S3 — Analyses of c-di-GMP amount by LC-mass spectroscopy. (A) Planktonic PA14 cells. (B) Biofilm PA14 cells. Data show the peaks for synthetic c-di-GMP (BIOLOGY Life Science Institute, Bermen, Germany), ethanol extract of PA14 without ginger addition (control), and ethanol extract of PA14 cultured with 1% ginger extract. c-di-GMP levels measured after about 7 minutes of retention time. The concentration of c-di-GMP was analyzed by calculating the area of peaks corresponding to the retention time. (TIF) [file pone.0076106.s003.tif]

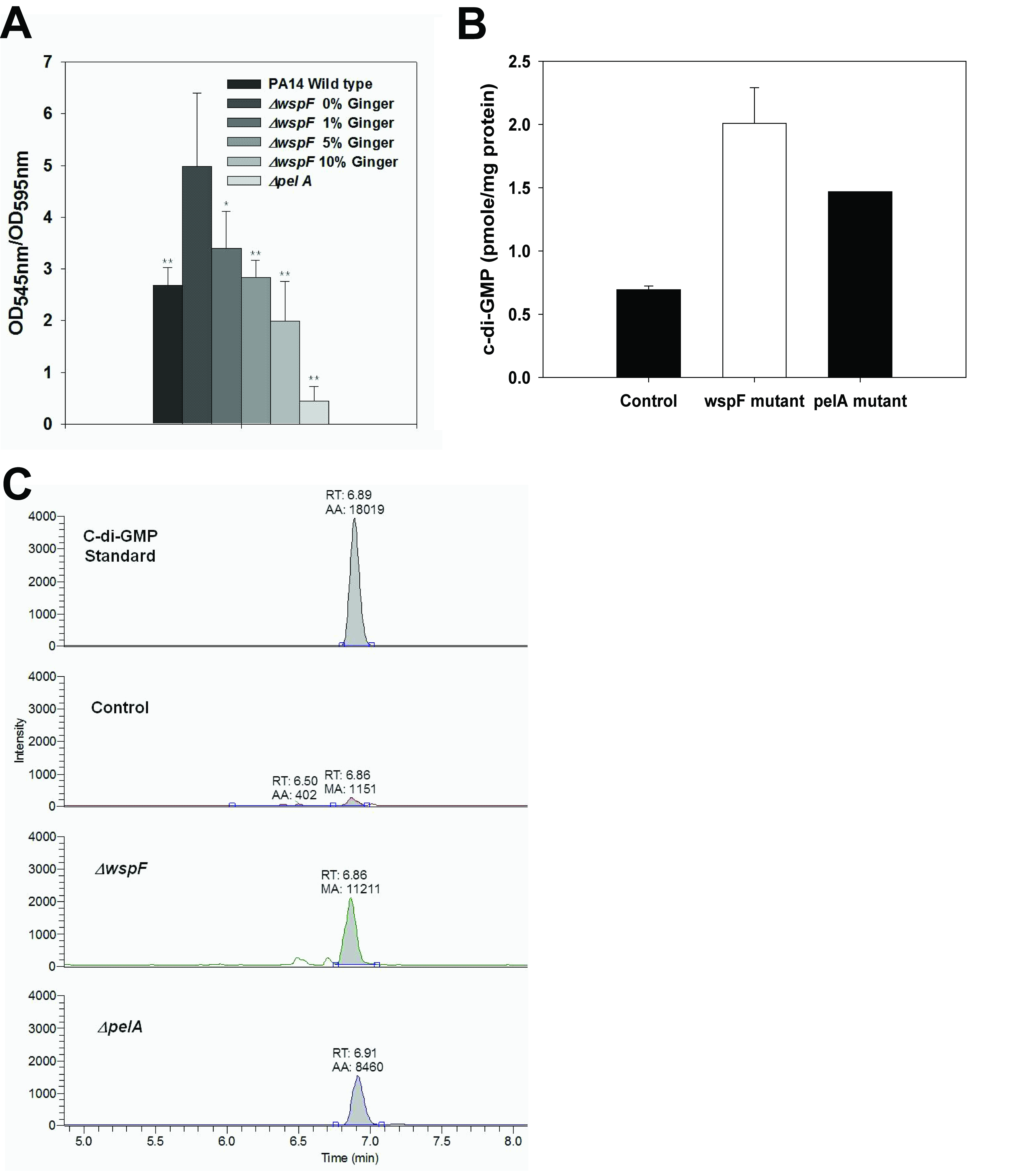

Supplement: Figure S4 — The effects of ginger extract on a PA14 mutant overproducing c-di-GMP (ΔwspF). (A) Quantification of biofilm formed in the wells of microtiter plates for ΔwspF cultures with various quantities of ginger extract (0, 1, 5, and 10%). The biofilm was quantified at 24 h of incubation by dividing OD at 545 nm by OD at 595 nm for cells stained with crystal violet. Error bars indicate the standard deviations of 6 measurements. *, P < 0.05 versus ΔwspF (0% ginger). **, P < 0.001 versus ΔwspF (0% ginger). (B) Concentration of c-di-GMP for control, ΔwspF, and ΔpelA. Error bars indicate the standard deviations of 3 measurements. *, P<0.001 versus the control. (C) Actual LC-MS peaks of c-di-GMP for control, ΔwspF, and ΔpelA. ΔpelA is a PA14 mutant forming no pellicle. (TIF) [file pone.0076106.s004.tif]
